# Supplementary material for: Unveiling the multifaceted potential of Pseudomonas khavaziana strain SR9: a promising biocontrol agent for wheat crown rot
Source: Microbiol Spectr. 2024 Aug 20;12(10):e00712-24. doi: 10.1128/spectrum.00712-24 (PMC11448100; doi:10.1128/spectrum.00712-24)
Supplement: Supplemental figures and tables — Fig. S1 to S7; Tables S1 to S6. [file spectrum.00712-24-s0001.docx]

Supplementary Material

Unveiling the multifaceted potential of *Pseudomonas khavaziana* strain SR9: a promising biocontrol agent for Wheat Crown Rot

Shengzhi Guo^1^, Yuqi Liu^1^, Yanling Yin^1,2^, Yating Chen^1^, Siyu Jia^1^, Tong Wu^1^, Jun Liao^1^, Xinyan Jiang^1^, Hafiz Abdul Kareem^1^, Xuejun Li^3^, Junfeng Pan^1#^, Yao Wang^1#^, Xihui Shen^1,2^

^1^State Key Laboratory for Crop Stress Resistance and High-Efficiency Production, Shaanxi Key Laboratory of Agricultural and Environmental Microbiology, College of Life Sciences, Northwest A&F University, Yangling, Shaanxi 712100, China

^2^Xinjiang Production and Construction Crops Key Laboratory of Protection and Utilization of Biological Resources in Tarim Basin, College of Life Sciences, Tarim University, Alar, Xinjiang 843300, China

^3^State Key Laboratory for Crop Stress Resistance and High-Efficiency Production, College of Agronomy, Northwest A&F University, Yangling, Shaanxi 712100, China

**#** **Address correspondence to:**

Jun-Feng Pan ([panjf@nwsuaf.edu.cn](mailto:panjf@nwsuaf.edu.cn))

and Yao Wang ([wangyao@nwsuaf.edu.cn](mailto:wangyao@nwsuaf.edu.cn))

Shengzhi Guo and Yuqi Liu contributed equally to this work.

Keywords: *Fusarium pseudograminearum*, *Pseudomonas khavaziana*, biological control, wheat crown rot, genomic analysis.

**RESULT**


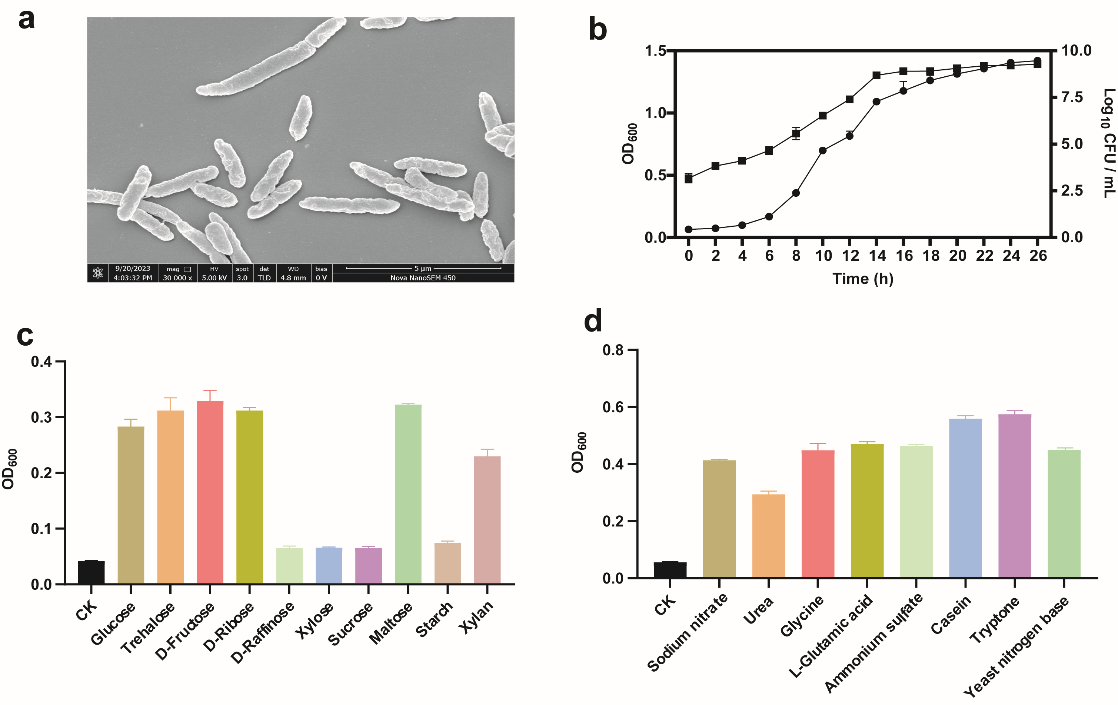


**Fig. S1 Carbon and nitrogen source utilization, colony morphology, and growth curve analysis of strain SR9.** (a): SEM morphology of the colonies of strain SR9 on LB agar plates after 24 hours of incubation; (b): Growth curve analysis of strain SR9 in LB medium. The OD_600_ and colony counts were measured at different time points, The squares represent the colony counts of strain SR9 at different time points, while the circles indicate the optical density (OD_600_) readings at those same time points. (c, d) Carbon/nitrogen source utilization of strain SR9 in carbon/nitrogen-free broth supplemented with different carbon sources (c) / nitrogen sources (d). The optical density at 600 nm (OD_600_) represents the growth status of strain SR9; Data are mean ± SD from three biological replicates.


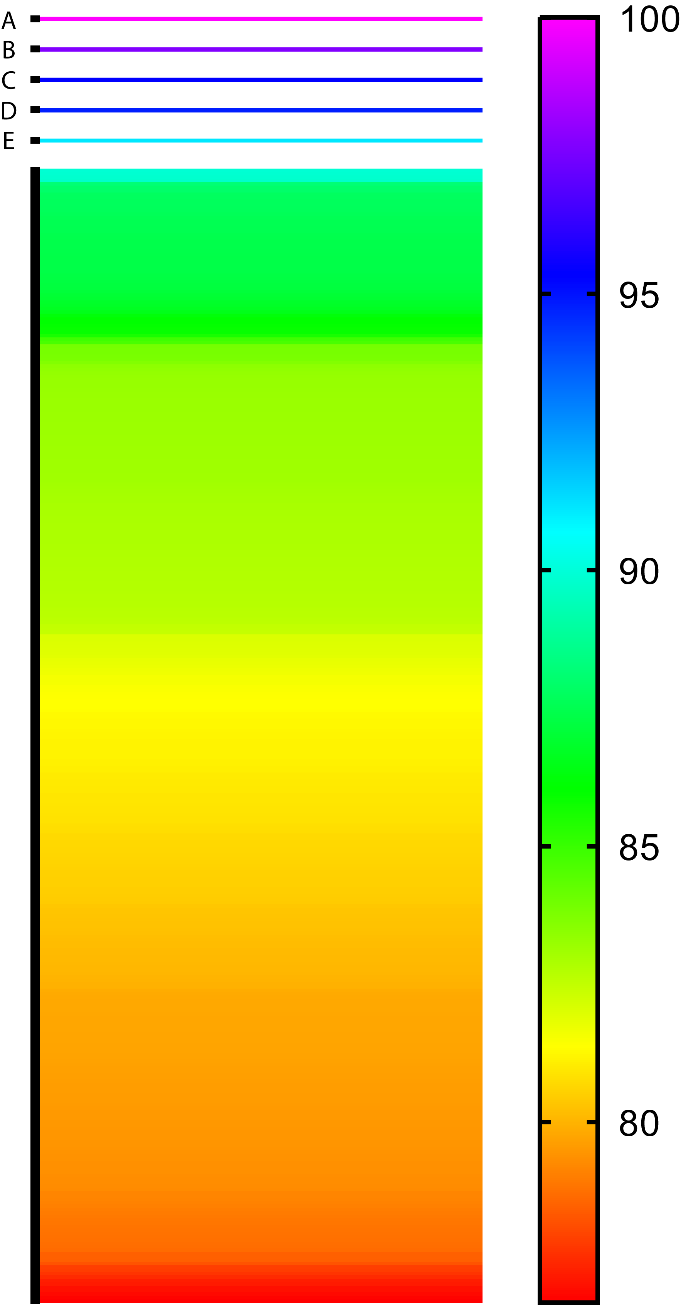


**Fig. S2 Average Nucleotide Identity analysis of related strains to SR9.** FastANI was used to determine the average nucleotide identity of selected species within the genus *Pseudomonas*. GraphPad Prism 9 is used for visualization. 1,A: SR9; 2,B: *P.khavaziana* SWRI124^T^; 3,C: *P.synxantha* DSM 18928^T^; 4,D: *P.libanensis* DSM 17149^T^; 5,E: *P.haemolytica* DSM 108987^T^; The following colors represent the ANI comparison of strain SR9 with other different type strains. For more, see the Sup Data1.


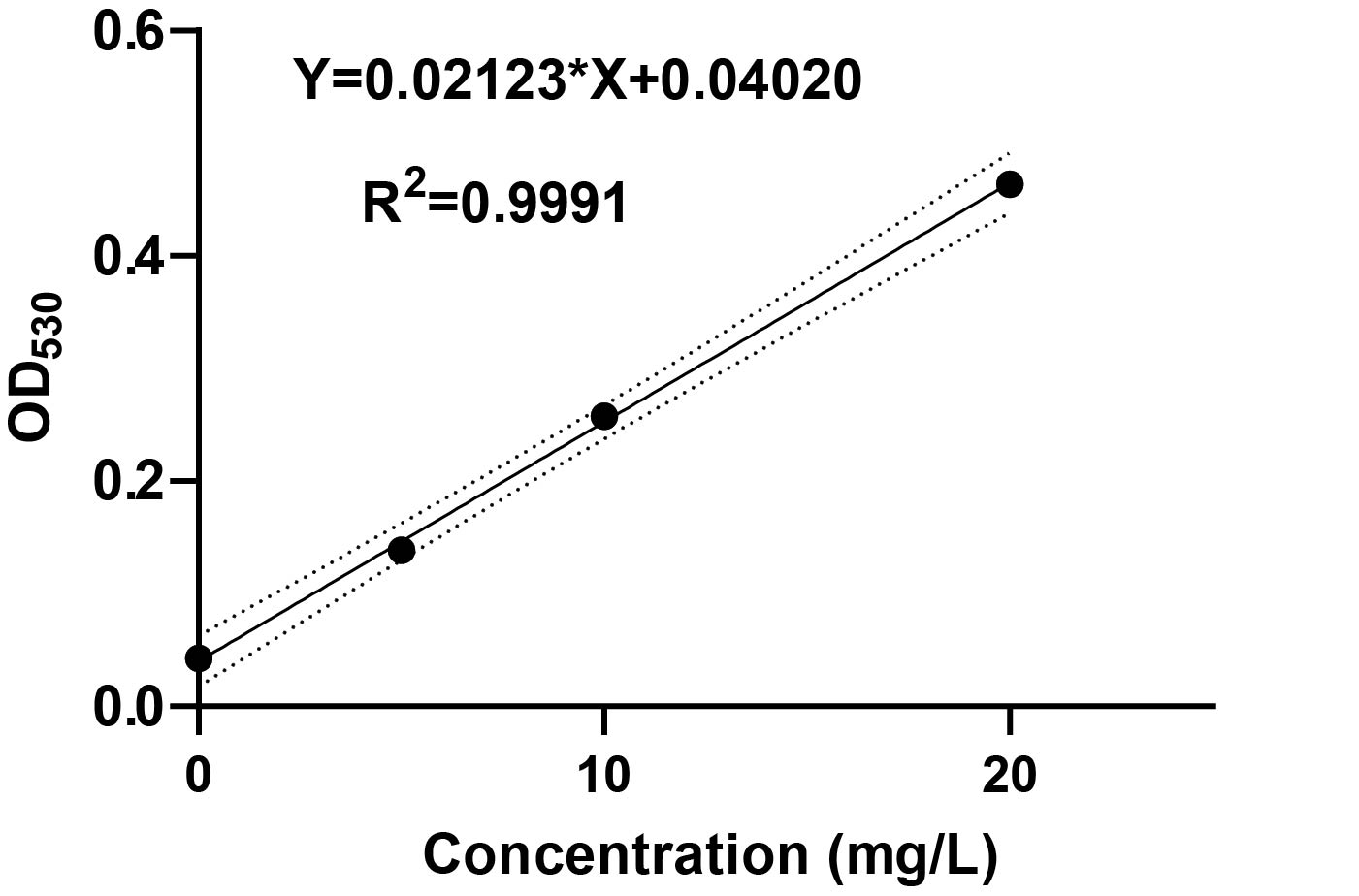


**Fig. S3 Standard curve for the quantification of indole-3-acetic acid (IAA) produced by strain SR9.** The concentration of IAA was determined by the colorimetric method using Salkowski reagent. The absorbance at 530 nm was measured and plotted against the known concentration of IAA. The equation of the linear regression and the correlation coefficient (R^2^) are shown in the figure.


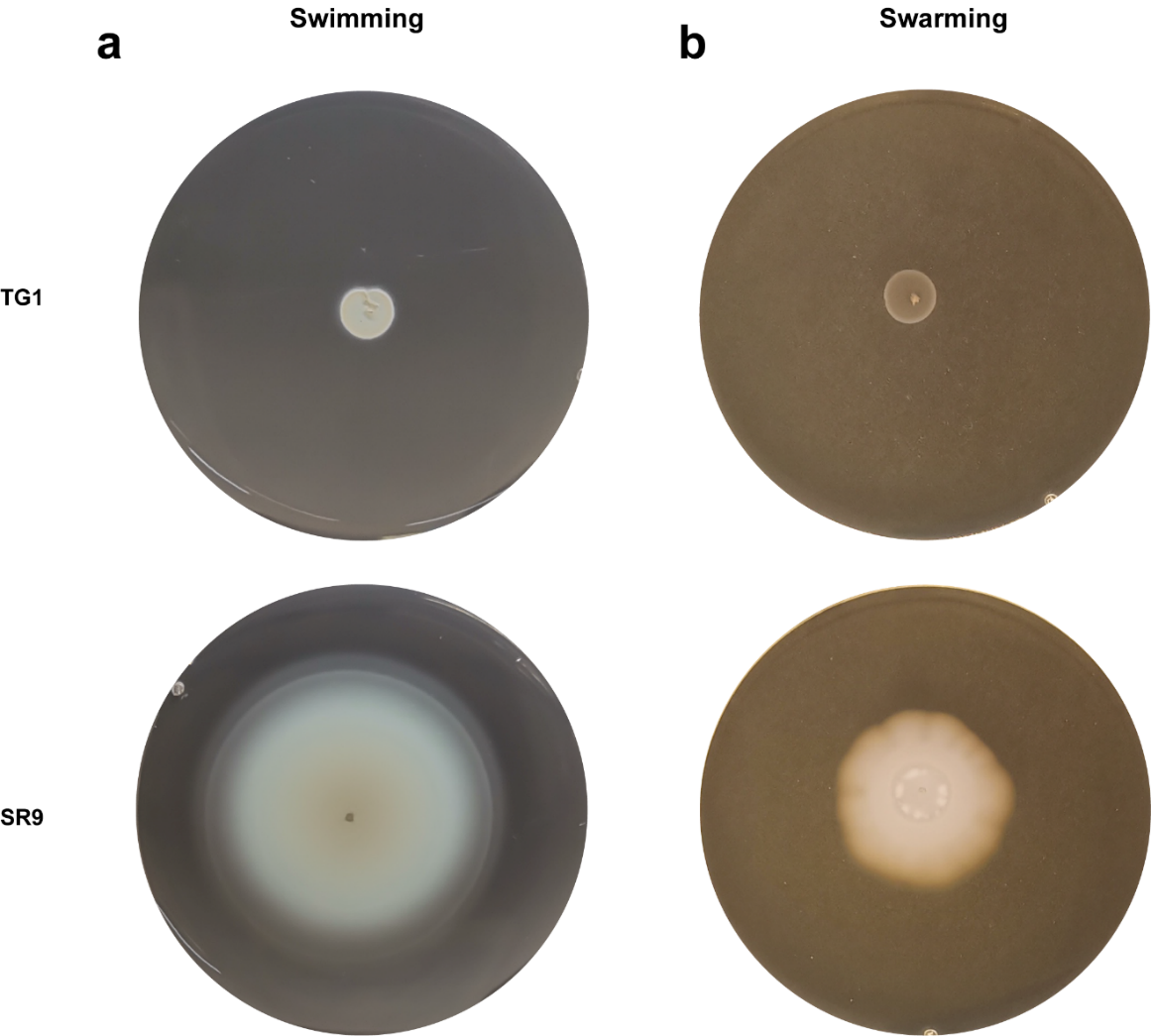


**Fig. S4 Motility of strain SR9.** (a) Swimming motility was assessed by inoculating strain SR9 into LB broth containing 0.25% agar and incubating at 30°C for 24 hours. (b) Swarming motility was assessed by inoculating strain SR9 onto LB agar plates containing 0.5% agar and 0.1% casein amino acid and incubating at 30°C for 24 hours. *Escherichia coli* TG1 strain used as a negative control.


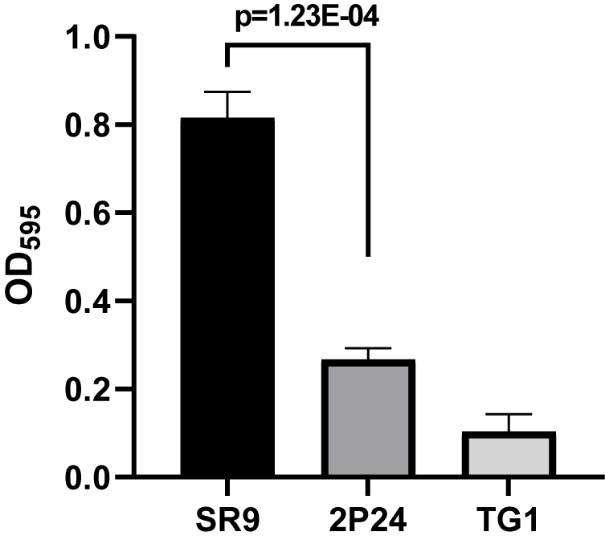


**Fig. S5 Biofilm adhesion of strain SR9.** Biofilm quantification of strain SR9 by crystal violet staining. 2P24 represents *P. fluorescens* 2P24; TG1 represents *Escherichia coli* TG1; Data are mean ± SD from three biological replicates.


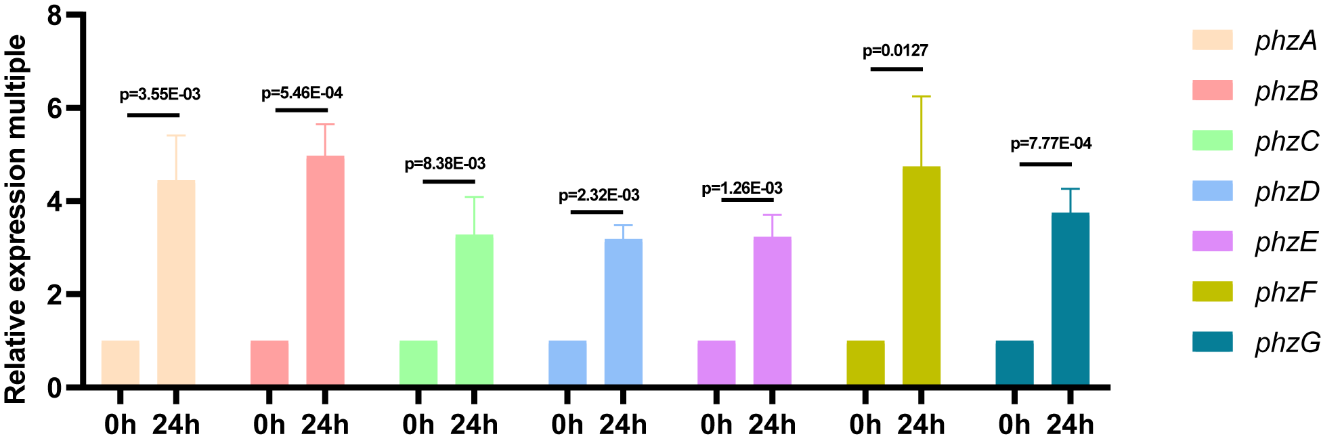
**Fig. S6 Fold expression of each gene in the phenazine gene cluster at 24 h during co-culture.** Data are presented as mean ± SD of three replicates.


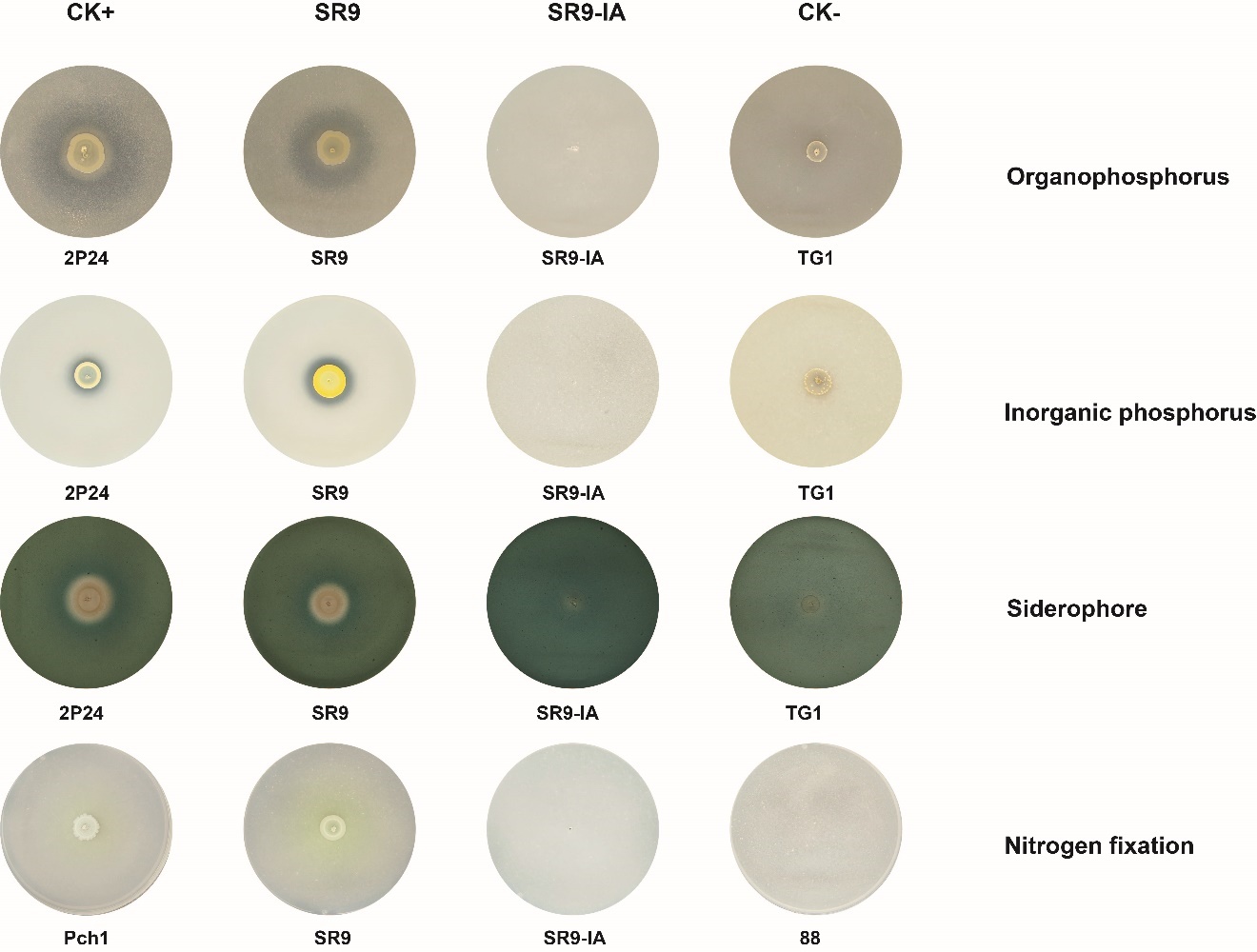


**Fig. S7 Functional characteristics of strain SR9.** Solubilization of organic and inorganic phosphorus, production of siderophores and nitrogen fixation of strain SR9. 2P24 represents *P. fluorescens* 2P24; SR9-IA represents the prepared strain SR9 suspension was inactivated; Pch1 represents *Sinorhizobium fredii* Pch1; TG1 represents *Escherichia coli* TG1; and 88 represents *Ruicaihuangia caeni* 88.

**Table S1 Comparative genome properties of strain SR9 with other *Pseudomonas* species.**

| **Strains** | **Genome size (Mbp)** | **GC (%)** | **Coding sequences** | **RNAs** | **GenBank Accession no.** |
| --- | --- | --- | --- | --- | --- |
| SR9 | 6.34 | 59.9 | 5693 | 87 | CP129946 |
| SWRI124^a^ | 6.48 | 59.6 | 6082 | 65 | GCA_019145205.1 |
| DSM 18928^b^ | 6.79 | 59.7 | 6323 | 72 | GCA_001439725.1 |
| DSM17149^c^ | 6.21 | 60.2 | 5639 | 63 | GCA_001439685.1 |
| WCS417^d^ | 6.17 | 60.3 | 5677 | 74 | GCA_000698265.1 |
| DSM 17152^e^ | 6.51 | 60.4 | 6040 | 66 | GCA_001983165.1 |
| CHA0^f^ | 9.87 | 63.4 | 6259 | 84 | GCA_900560965.1 |
| 2P24^g^ | 6.61 | 60.8 | 6194 | 81 | GCA_002865505.1 |
| UASWS1668^h^ | 6.17 | 60.3 | 5645 | 68 | GCA_008693205.1 |
| S58^i^ | 6.49 | 61.1 | 6012 | 82 | GCA_009818265.1 |
| SCA7^j^ | 6.78 | 59.1 | 6288 | 92 | GCA_018138145.1 |
| 923^k^ | 5.76 | 64.3 | 5260 | 100 | GCA_023065975.1 |
| KT2440^l^ | 6.18 | 61.5 | 5715 | 96 | GCA_000007565.2 |

^a^represents *P.khavaziana* SWRI124; ^b^represents *P.synxantha* DSM 18928; ^c^represents *P.libanensis* DSM17149; ^d^represents *P.simiae* WCS417; ^e^represents *P.gessardii* DSM 17152; ^f^represents *P.protegens* CHA0; ^g^represents *P.fluorescens* 2P24; ^h^represents *P.koreensis* UASWS1668; ^i^represents *P.mediterranea* S58; ^g^represents *P.sp.*SCA7; ^k^represents *P.mosselii* 923; ^l^represents *P.putida* KT2440;

**Table S2. Antimicrobial susceptibility test of *P. khavaziana* SR9.**

| **Antibiotics(µg/mL)** | **0** | **5** | **10** | **20** | **50** | **100** |
| --- | --- | --- | --- | --- | --- | --- |
| Ampicillin | + | + | + | + | + | + |
| Kanamycin | + | + | + | + | + | - |
| Chloramphenicol | + | + | + | + | + | + |
| Nalidixic acid | + | + | + | + | + | + |
| Ceftazidime | + | + | + | + | + | + |
| Rifampicin | + | + | - | - | - | - |
| Ciprofloxacin | + | - | - | - | - | - |
| Tetracycline | + | - | - | - | - | - |
| Gentamicin | + | - | - | - | - | - |

‘+’ represents that the strain SR9 can grow in the liquid medium under this concentration of antibiotics, ‘–’ represents not.

**Table S3. Genes related to antagonistic fungi.**

| Classification | Gene ID | Length (bp) | Product |
| --- | --- | --- | --- |
| Chitin-related | gene3973 | 1065 | Chitinase (EC 3.2.1.14) |
|  | gene3974 | 636 | Chitin binding protein |
| Murein hydrolase-related | gene511 | 1296 | Murein hydrolase activator EnvC |
|  | gene1504 | 837 | Murein hydrolase activator NlpD |
|  | gene2545 | 1542 | cell wall degradation protein |
| Cellulose-related | gene2345 | 1092 | Cellulase M and related proteins |

**Table S4. Primers for *phzE* gene knockout,complement, and quantification.**

| Primer name | Sequence (5'to 3') | Product |
| --- | --- | --- |
| *phzF*_up_ F_*EcoR*Ⅰ | *GGAAACAGCTATGACATGATTAC*GAATTCCAGCCAGCAACGGCAACA | PCR primers amplify *phzF*upstream fragment |
| *phzF*_up_R | **CGGTAAATCATCGGCGTCA** |  |
| *phzF*_down_F | **TGACGCCGATGATTTACCG**ACGCGAGTCGAGGTATCAGGTA | PCR primers amplify *phzF* downstream fragment |
| *phzF*_down_R_*BamH*Ⅰ | *GCATGCCTGCAGGTCGACTCTAGA*GGATCCGCTACTCAGGGCTGCAAACG |  |
| *PhzF*_F*_Xho*Ⅰ | CCGCTCGAGCATGCACAACTACGTCATTATCGACGC | To generate  pBBR1-MCS5-*phzF* |
| *PhzF*_R*_EcoR*Ⅰ | CCGGAATTCTCATAGCACGATGGTCCCCCGT |  |
| RT_*phzF*_F | GTGTTCTTTGACGCCGATGATTTAC | Quantitative primer amplification of *phzF* fragment |
| RT_*phzF*_R | GATGACGCTGCCGTTCTGG |  |
| RT_16S_F | AACACCAGTGGCGAAGGC | Quantitative primer amplification of 16S rRNA fragments |
| RT_16S_R | ACTTAATGCGTTAGCTGCGCC |  |

Red letters indicate restriction enzyme cutting sites added for cloning. Letters in boldface denote the annealing regions for overlap PCR. Italic letters indicate homology arm regions with vector pK18*mobsacB*.

**Table S5. Genes related to plant nutrition and growth.**

| Classification/Pathway | Gene ID | Length(bp) | Product |
| --- | --- | --- | --- |
| Phosphorus Solubilization-related | gene1502 | 750 | Exopolyphosphatase (EC 3.6.1.11) |
|  | gene5646 | 1503 | Exopolyphosphatase (EC 3.6.1.11) |
|  | gene5182 | 528 | Inorganic pyrophosphatase (EC 3.6.1.1) |
|  | gene992 | 1542 | Phosphodiesterase/alkaline phosphatase D |
|  | gene1698 | 582 | Alkaline phosphatase (EC 3.1.3.1) |
|  | gene2038 | 1572 | Alkaline phosphatase D |
|  | gene5564 | 1947 | Alkaline phosphatase isozyme conversion protein |
|  | gene870 | 1701 | Acid phosphatase (EC 3.1.3.2) |
|  | gene227 | 423 | Phosphonate ABC transporter phosphate-binding periplasmic component (TC 3.A.1.9.1) |
|  | gene1837 | 285 | Phosphonate ABC transporter phosphate-binding periplasmic component (TC 3.A.1.9.1) |
|  | gene2197 | 309 | Phosphonate ABC transporter phosphate-binding periplasmic component (TC 3.A.1.9.1) |
|  | gene5167 | 270 | Phosphonate ABC transporter phosphate-binding periplasmic component (TC 3.A.1.9.1) |
| Nitrogen metabolism-related | gene519 | 1437 | Nitrogen regulation protein NR(I), GlnG (=NtrC) |
|  | gene520 | 1086 | Nitrogen regulation protein NtrB (EC 2.7.13.3) |
|  | gene1067 | 273 | Phosphocarrier protein, nitrogen regulation associated |
|  | gene1069 | 465 | PTS IIA-like nitrogen-regulatory protein PtsN |
|  | gene5558 | 2280 | FIG001592: Phosphocarrier protein kinase/phosphorylase, nitrogen regulation associated |
|  | gene5685 | 339 | Nitrogen regulatory protein P-II, GlnK |
| Spermidine biosynthesis | gene804 | 1914 | Biosynthetic arginine decarboxylase (EC 4.1.1.19) |
|  | gene998 | 1164 | Pyridoxal 5-phosphate (PLP)-dependent ornithine decarboxylase (EC 4.1.1.17) |
|  | gene5440 | 1191 | S-adenosylmethionine synthetase (EC 2.5.1.6) |
|  | gene473 | 1107 | Agmatine deiminase (EC 3.5.3.12) |
|  | gene1700 | 1407 | Homospermidine synthase (EC 2.5.1.44) |
|  | gene1910 | 756 | Spermidine synthase-like protein |
|  | gene2533 | 1245 | Carboxynorspermidine synthase (EC 1.5.1.43) |
| IAA biosynthesis | gene4798 | 792 | Aliphatic amidase AmiE (EC 3.5.1.4) |
|  | gene5331 | 795 | 5-aminopentanamidase (EC 3.5.1.30) / Aliphatic amidase AmiE (EC 3.5.1.4) |
|  | gene1058 | 849 | FIG003879: Uncharacterized subgroup of the nitrilase superfamily |
|  | gene2663 | 921 | Transcriptional regulator in custer with plant-induced nitrilase |
|  | gene2664 | 927 | Plant-induced nitrilase (EC 3.5.5.1), hydrolyses beta-cyano-L-alanine |
|  | gene5525 | 1428 | Aldehyde dehydrogenase (EC 1.2.1.3); Probable coniferyl aldehyde dehydrogenase (EC 1.2.1.68) |
|  | gene5416 | 1473 | Betaine aldehyde dehydrogenase (EC 1.2.1.8) |

**Table S6. Genes related to encoding flagella and chemotaxis.**

| Classification | Gene ID | Length (bp) | Product |
| --- | --- | --- | --- |
| Chemotaxis-related | gene701 | 1935 | Methyl-accepting chemotaxis protein |
|  | gene1421 | 1623 | Methyl-accepting chemotaxis protein |
|  | gene1874 | 1482 | Methyl-accepting chemotaxis protein |
|  | gene2022 | 1968 | Methyl-accepting chemotaxis protein |
|  | gene2316 | 1215 | Methyl-accepting chemotaxis protein |
|  | gene2388 | 1944 | Methyl-accepting chemotaxis protein |
|  | gene3191 | 1494 | Methyl-accepting chemotaxis protein |
|  | gene5486 | 2052 | Methyl-accepting chemotaxis protein |
|  | gene4257 | 1566 | Aerotaxis sensor receptor protein |
|  | gene1422 | 513 | CheW domain protein WspB |
|  | gene2321 | 540 | Positive regulator of CheA protein activity (CheW) |
|  | gene4112 | 456 | Positive regulator of CheA protein activity (CheW) |
|  | gene4113 | 792 | CheW domain protein |
|  | gene1153 | 903 | Chemotaxis protein CheV (EC 2.7.3.-) |
|  | gene4106 | 936 | Chemotaxis protein CheV (EC 2.7.3.-) |
|  | gene4442 | 933 | Chemotaxis protein CheV (EC 2.7.3.-) |
|  | gene1425 | 2268 | Signal transduction histidine kinase CheA |
|  | gene2319 | 2040 | Signal transduction histidine kinase CheA |
|  | gene4118 | 2217 | Signal transduction histidine kinase CheA |
|  | gene5485 | 5814 | Chemotaxis protein cheA (EC 2.7.3.-) |
|  | gene1207 | 696 | Signal Transduction Histidine Kinase (STHK) with CheB and CheR activity |
|  | gene1426 | 1011 | Chemotaxis response regulator protein-glutamate methylesterase CheB (EC 3.1.1.61) |
|  | gene1675 | 4146 | Multidomain signal transduction protein including CheB-like methylesterase, CheR-like methyltransferase and BaeS-like histidine kinase |
|  | gene2324 | 1080 | Chemotaxis response regulator protein-glutamate methylesterase CheB (EC 3.1.1.61) |
|  | gene4117 | 1137 | Chemotaxis response regulator protein-glutamate methylesterase CheB (EC 3.1.1.61) |
|  | gene2322 | 810 | Chemotaxis protein methyltransferase CheR (EC 2.1.1.80) |
|  | gene2993 | 816 | MCP methyltransferase, CheR-type |
|  | gene4441 | 828 | Chemotaxis protein methyltransferase CheR (EC 2.1.1.80) |
|  | gene2323 | 534 | Chemotaxis protein CheD |
|  | gene2317 | 369 | Chemotaxis regulator - transmits chemoreceptor signals to flagellar motor components CheY |
|  | gene4120 | 387 | Chemotaxis regulator - transmits chemoreceptor signals to flagellar motor components CheY |
|  | gene4119 | 789 | Chemotaxis response - phosphatase CheZ |
| Flagella-related | gene4123 | 1317 | Flagellar biosynthesis protein FlhF |
|  | gene4124 | 2133 | Flagellar biosynthesis protein FlhA |
|  | gene4131 | 1137 | Flagellar biosynthesis protein FlhB |
|  | gene4132 | 786 | Flagellar biosynthesis protein FliR |
|  | gene4133 | 270 | Flagellar biosynthesis protein FliQ |
|  | gene4134 | 744 | Flagellar biosynthesis protein FliP |
|  | gene4135 | 438 | Flagellar biosynthesis protein FliO |
|  | gene4153 | 390 | Flagellar biosynthesis protein FliS |
|  | gene4445 | 471 | Flagellar biosynthesis protein FlgN |
|  | gene4148 | 330 | Flagellar hook-basal body complex protein FliE |
|  | gene4147 | 1776 | Flagellar M-ring protein FliF |
|  | gene4146 | 1017 | Flagellar motor switch protein FliG |
|  | gene4145 | 765 | Flagellar assembly protein FliH |
|  | gene4144 | 1359 | Flagellum-specific ATP synthase FliI |
|  | gene4143 | 450 | Flagellar protein FliJ |
|  | gene4139 | 1308 | Flagellar hook-length control protein FliK |
|  | gene4138 | 504 | Flagellar basal body-associated protein FliL |
|  | gene5626 | 408 | Similar to flagellar basal body-associated protein FliL |
|  | gene4137 | 969 | Flagellar motor switch protein FliM |
|  | gene4136 | 468 | Flagellar motor switch protein FliN |
|  | gene4443 | 696 | Flagellar basal-body P-ring formation protein FlgA |
|  | gene4440 | 408 | Flagellar basal-body rod protein FlgB |
|  | gene4163 | 786 | Flagellar basal-body rod protein FlgG |
|  | gene4164 | 741 | Flagellar basal-body rod protein FlgF |
|  | gene4438 | 720 | Flagellar basal-body rod modification protein FlgD |
|  | gene4439 | 444 | Flagellar basal-body rod protein FlgC |
|  | gene4437 | 1317 | Flagellar hook protein FlgE |
|  | gene4162 | 696 | Flagellar L-ring protein FlgH |
|  | gene4161 | 1107 | Flagellar P-ring protein FlgI |
|  | gene4160 | 1260 | Flagellar protein FlgJ [peptidoglycan hydrolase] |
|  | gene4159 | 2046 | Flagellar hook-associated protein FlgK |
|  | gene4444 | 324 | Negative regulator of flagellin synthesis FlgM (anti-sigma28) |
|  | gene427 | 795 | L-cystine ABC transporter (wide substrate range), substrate-binding protein FliY |
|  | gene684 | 852 | Flagellar motor rotation protein MotA |
|  | gene685 | 1020 | Flagellar motor rotation protein MotB |
|  | gene1941 | 330 | Uncharacterized homolog of the cytoplasmic domain of flagellar protein FhlB |
|  | gene4115 | 888 | Flagellar motor rotation protein MotB |
|  | gene4116 | 741 | Flagellar motor rotation protein MotA |
|  | gene4121 | 741 | RNA polymerase sigma factor for flagellar operon |
|  | gene4122 | 834 | Flagellar synthesis regulator FleN |
|  | gene4149 | 1377 | Flagellar two-component response regulator FleR |
|  | gene4150 | 1212 | Flagellar sensor histidine kinase FleS |
|  | gene4151 | 1473 | Flagellar regulatory protein FleQ |
|  | gene4154 | 1368 | Flagellar cap protein FliD |
|  | gene4446 | 750 | Flagellar brake protein YcgR |
